# A Curvature-Mediated Mechanism for Localization of Lipids to Bacterial Poles

**DOI:** 10.1371/journal.pcbi.0020151

**Published:** 2006-11-10

**Authors:** Kerwyn Casey Huang, Ranjan Mukhopadhyay, Ned S Wingreen

**Affiliations:** 1 Department of Molecular Biology, Princeton University, Princeton, New Jersey, United States of America; 2 Department of Physics, Clark University, Worcester, Massachusetts, United States of America; Weill Medical College of Cornell University, United States of America

## Abstract

Subcellular protein localization is a universal feature of eukaryotic cells, and the ubiquity of protein localization in prokaryotic species is now acquiring greater appreciation. Though some targeting anchors are known, the origin of polar and division-site localization remains mysterious for a large fraction of bacterial proteins. Ultimately, the molecular components responsible for such symmetry breaking must employ a high degree of self-organization. Here we propose a novel physical mechanism, based on the two-dimensional curvature of the membrane, for spontaneous lipid targeting to the poles and division site of rod-shaped bacterial cells. If one of the membrane components has a large intrinsic curvature, the geometrical constraint of the plasma membrane by the more rigid bacterial cell wall naturally leads to lipid microphase separation. We find that the resulting clusters of high-curvature lipids are large enough to spontaneously and stably localize to the two cell poles. Recent evidence of localization of the phospholipid cardiolipin to the poles of bacterial cells suggests that polar targeting of some proteins may rely on the membrane's differential lipid content. More generally, aggregates of lipids, proteins, or lipid-protein complexes may localize in response to features of cell geometry incapable of localizing individual molecules.

## Synopsis

Bacteria contain proteins that localize to different regions of the cell—the poles, the middle of the cell, or along the membrane in specific structures such as helices. This localization is often critical to function, from division-site placement, to cell-cycle progression, to maintaining the shape of the cell. How proteins localize to the poles of cells varies, and the mechanism is unknown for many proteins. A surprising discovery in recent years is that the membrane component cardiolipin localizes to the poles of the rod-shaped bacteria Escherichia coli and Bacillus subtilis. This work of Huang, Mukhopadhyay, and Wingreen presents a model of lipid polar localization in which clusters of cardiolipin are formed naturally due to the constraint of the membrane by the rigid cell wall, and these clusters are large enough to localize to the poles of the cell due to curvature, suggesting that clusters of lipids may serve to recruit proteins to the poles.

## Introduction

In the past decade, intracellular fluorescence microscopy has fashioned a new appreciation of protein organization and segregation on bacterial membranes. From polar localization of developmental proteins such as DivJ, PleC, and DivK in Caulobacter crescentus [1,2], to septal localization of the divisome [3,4], to helical conformations of the cytoskeletal elements such as MreB [5], the list penetrates nearly every manner of protein function and bacterial species. In each instance, protein localization requires the breaking of the cell's symmetry. In the rod-shaped bacterium *Escherichia coli,* the Min-protein system targets the poles dynamically based on self-organized pole-to-pole oscillations [6]. This spontaneous spatiotemporal symmetry breaking remains one of the few examples of membrane localization that does not rely on the presence of an anchor, whose targeting mechanism remains unknown, but protein oscillations have not been revealed as a common mechanism for polar localization in bacteria. The possibility that the membrane itself is differentially organized has been relatively unexplored. In the first in vivo observation of spatial heterogeneity of bacterial phospholipids, it was recently shown, using the cardiolipin-specific staining agent nonyl acridine orange (NAO), that the phospholipid cardiolipin localizes to the polar and septal regions of the cytoplasmic membrane of E. coli [7,8] and Bacillus subtilis [9]. Furthermore, significantly enhanced levels of cardiolipin were found in the membrane of E. coli minicells [10] and in the engulfment and forespore membranes of B. subtilis cells during sporulation [9]. How spatial organization of lipids is established, and, in particular, how cardiolipin is directed to bacterial poles, is as yet an unanswered question. Here we propose an equilibrium mechanism based on lipid microphase separation to explain the observed polar localization of cardiolipin.

One possibility is that cardiolipin localization is driven purely by lipid phase segregation. Indeed, fluorophore-labeled phospholipids have been observed to phase segregate in giant unilamellar vesicles into micron-scale domains, often with different curvatures [11,12]. This in vitro phase segregation is driven in large part by line tension [13] assisted by the ordering agent cholesterol. However, fundamental differences exist between such large-scale phase segregation observed in model membranes and lipid localization in bacteria. In particular, the experimentally observed rapid repartitioning of cardiolipin to the division site [7–9] would be strongly disfavored if cardiolipin was segregated in a single, large cluster at one or both poles. Instead, we will show that the geometrical constraint of the (inner) plasma membrane by the cell wall naturally produces stable *finite*-sized lipid clusters which can spontaneously and independently target the two poles of the cell as well as the nascent division site.

In bacteria, the cell wall defines the overall shape of the membrane [14], but on the molecular scale the membrane shape is likely to be influenced by the different chemical structures of bacterial phospholipids. The difference in curvature between the poles and the cylindrical region in between is often invoked to explain the polar localization of proteins [15]. In a similar fashion, it is natural to expect for structural reasons that, in the inner leaflet of the cytoplasmic membrane bilayer, a lipid with a headgroup cross-sectional area much smaller than that of its lipid tail will be attracted to the high curvature of the poles. Of the three dominant bacterial lipids (cardiolipin, phosphatidylglycerol, and phosphatidylethanolamine), cardiolipin is the most likely to seek high curvature based on head-to-tail ratio, and preliminary experiments have shown that two-component giant unilamellar vesicles containing cardiolipin frequently adopt morphologies indicative of highly curved components. However, the difference in length scales between single lipid molecules and the radius of curvature of the cell membrane results in ineffectually small targeting energies; for a micron-size bacteria and a single lipid molecule with intrinsic radius of curvature in the tens of nanometers, a rough estimate of the energy difference between polar and nonpolar localization is approximately 1% of the thermal energy *k_B_T* (see Materials and Methods). In the presence of thermal fluctuations, it is therefore highly unlikely that single, independent lipids will segregate in the membrane.

**Figure 1:**
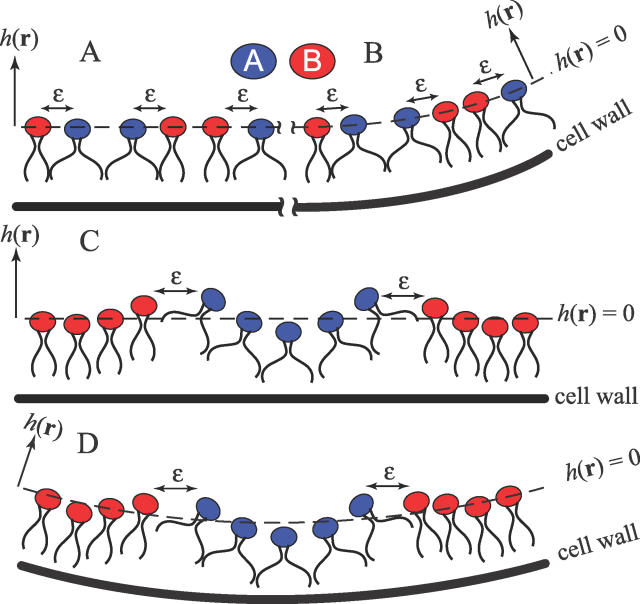
Schematic of Lipid Clustering in Different Membrane Configurations (A) A homogeneous mixture of two lipid components (type A in blue, B in red) has a high energy because of the abundance of unlike nearest-neighbor pairs, each contributing an energy ε (see Materials and Methods). (B) Same as (A), except the cell wall has higher curvature; this results in a slight reduction in the energy of lipids of type A, which have higher intrinsic curvature. (C) A cluster of lipids of type A is accompanied by membrane curvature (∇^2^ *h*(**r**)). Though the elastic energy of the membrane increases relative to (A), the formation of clusters eliminates unlike nearest-neighbor pairs and so reduces the total energy. (D) The same configuration in (C) shifted to a region of cell wall with higher curvature experiences a large reduction in energy, leading to cluster localization at the poles.

The analysis above is critically altered by including the interactions among lipids that can lead to phase segregation (see Figure 1). We show that local (short-range) attractive chemical interactions (possibly mediated by divalent counterions [9]) can induce lipids in bacterial cells to form clusters, limited in size by long-range elastic interactions. These finite lipid clusters can stably target the poles based on their enhanced sensitivity as an aggregate to small variations in curvature. The lipid-raft model of cholesterol and sphingolipid in the outer leaflet of eukaryotic cell membranes proposes that ordered domains floating in a liquid bilayer act as signaling platforms that couple extracellular events to pathways inside the cell [16,17]. In this report, we propose a mechanism for lipid-cluster formation within the inner leaflet of bacterial membranes that leads directly to polar localization. The self-organizing nature of this mechanism potentially points to a similar origin of lipid domains in eukaryotic cells, and suggests that bacterial lipid clusters could have similar utility to lipid rafts as a stage for targeting proteins involved in a wide variety of biological processes.

## Results

### Cell Wall Constraint Leads to Finite-Sized Lipid Clusters

We first address how the presence of the cell wall affects the energetics of the membrane and mediates the formation of lipid clusters. We divide the total energy of the inner leaflet of a bacterial membrane composed of two components (e.g*.*, cardiolipin and phosphatidylethanolamine) into two competing interactions: a short-range chemical attraction and a long-range elastic repulsion. On the one hand, a short-range, attractive interaction between like lipids has been shown theoretically to result in phase segregation [18], made kinetically possible by the high diffusibility of lipids in bilayers (*D* ∼ 10 *μ*m^2^/s) [19]. For the measured proportion of cardiolipin in E. coli membranes, such an interaction between cardiolipin molecules by itself would create a single large cluster covering roughly 5%–10% of the membrane [10,18]. (Note that such a cluster in a 4-micron bacterium with a 1-micron diameter would not cover even a single pole, which accounts for ∼12.5% of the membrane. Thus, it is unlikely that phase segregation alone will lead to approximately equal concentrations of cardiolipin at the two poles). In this paper, we will assume for simplicity that the attractive interaction occurs between immediately neighboring like lipids, but our conclusions do not depend on the precise form of this interaction.

On the other hand, if one of the membrane components (e.g., cardiolipin) has a large intrinsic curvature *γ,* cluster formation due to short-range interactions is counterbalanced by a cell-wall–induced elastic interaction between lipids. Conformations in which the curvature of the membrane is as extreme as the intrinsic curvature of the lipids (∼10nm) incur a penalty for separating the membrane away from the rigid cell wall; the net result is a repulsive long-range elastic potential *V*(*r*) between high-curvature lipids that makes overly large lipid clusters energetically unfavorable. We refer to the penalty for the mismatch between membrane curvature and lipid intrinsic curvature as the *stiffness* contribution to the free energy, with a magnitude depending on the stiffness modulus *κ,* and we call the penalty for membrane separation from the cell wall the *pinning* contribution, with pinning modulus *λ*. Although the cell-wall curvature has opposite effects on lipid molecules in the outer leaflet, it is well-established in both bacterial membranes and eukaryotic plasma membranes that the lipid compositions of the two leaflets can be very different [16,17], and we will focus on the properties of the inner leaflet [20,21].

**Figure 2:**
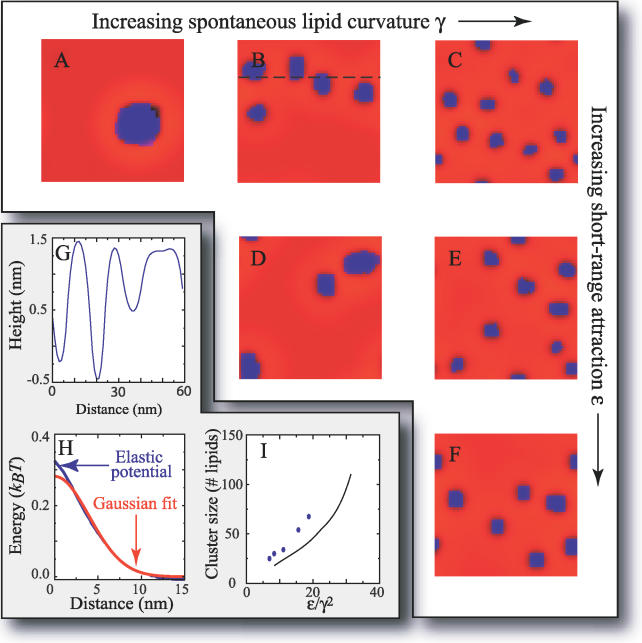
Lipid Cluster Size as a Function of Short-Range Attractive Interaction and Intrinsic Lipid Curvature (A–F) Representative distribution in a mixed membrane of lipids A and B, where lipid A has intrinsic curvature *γ,* lipid B has no intrinsic curvature, and immediately neighboring lipids of the same type experience an attractive interaction *ɛ*. The stiffness modulus (*κ* = 25 *k_B_T*), pinning modulus (*λ* = 0.25 *k_B_T*/nm^4^), and fraction of lipid A (*ϕ* = 0.075) are the same in all panels. Blue indicates the presence of lipid A, while red represents the height of the membrane relative to the cell wall. (A–C) *ɛ* = 2.5 *k_B_T,* and *γ* = 0.2 nm^−1^, 0.4 nm^−1^, 0.6 nm^−1^, respectively (D,E) *ɛ* = 3 *k_B_T,* and *γ* = 0.4 nm^−1^, 0.6 nm^−1^, respectively. (F) *ɛ* = 4 *k_B_T* and *γ* = 0.6 nm^−1^. (G) Height profile of the membrane along the dashed black line in (B). (H) The long-range repulsive elastic interaction between two lipids is well fit by a Gaussian. (I) Average cluster size as a function of *ɛ*/*γ* ^2^. Results of simulations are shown with blue dots, while the black line represents the theoretical prediction based on minimizing free energy per lipid.

Phase segregation in two-component bilayers, as seen in Figure 2A, occurs as a first-order phase transition with respect to the short-range attraction ɛ between like lipids [18]. The critical attraction strength *ɛ*_crit_ at which phase segregation occurs is lowest when the two lipid components each comprise 50% of the membrane, and increases as the lipid fractions become more unbalanced. Above *ɛ*_crit_ (i.e., in the regime of spontaneous phase segregation), the short-range attraction lowers the free energy by an amount that scales approximately linearly with the number of lipids in the cluster. In contrast, the slow increase in membrane deformation as additional high-intrinsic-curvature lipids are added to a cluster slowly increases the free energy, proportional to the square of the total number of lipids in the cluster. The growth of clusters therefore leads to a decrease in the free energy of the membrane until the clusters reach a preferred size determined by the minimum free energy per lipid, as shown schematically in Figure 1. We have assumed a fixed value for the membrane stiffness modulus *κ* = 25 *k_B_T* that roughly matches measured elastic moduli for lipid-bilayer dynamics [22,23], and we have used a pinning modulus *λ* = 0.25 *k_B_T*/nm^4^ to coordinate lipid cluster sizes with the size of our simulations.

In Figure 2A–2F, we plot the results of Monte Carlo simulations (see Materials and Methods) of a 60 × 60 periodic square lattice composed of two types of lipids, A and B, where the intrinsic curvature of the lipids is *γ* and 0, respectively, and the membrane composition is 7.5% lipid A and 92.5% lipid B. Lipids of type A are shown in blue, and superimposed in shades of red is the height of the membrane *h*(**r**) relative to the cell wall, which we initially take to be flat. *h*(**r**) = 0 corresponds to the balance point between osmotic pressure pushing the membrane out and the cell wall holding the membrane in. For fixed values *κ* = 25 *k_B_T* and *λ* = 0.25 *k_B_T*/nm^4^, the lipids phase segregate at large values of the short-range attraction *ɛ* and small values of intrinsic curvature *γ*. In Figure 2A, due to the small intrinsic curvature of lipid A, the elastic energy due to both curvature and pinning is insignificant compared with the short-range attraction between like lipids; thus the membrane minimizes total energy with a single cluster of lipid A and the resulting height profile is close to zero (i.e., the membrane is closely pinned to the cell wall). As the intrinsic curvature *γ* of lipid A increases from 0.2 nm^−1^ (A) to 0.4 nm^−1^ (B,D) to 0.6 nm^−1^ (C,E,F), the single cluster of lipid A breaks up into smaller clusters that organize into a semiregular lattice. This reduction in cluster size can be counteracted by increasing the short-range attraction *ɛ* from 2.5 *k_B_T* (A–C) to 3 *k_B_T* (D,E) to 4 *k_B_T* (F). A typical height profile *h*(**r**), along the dashed black line in Figure 2B, is shown in Figure 2G. Each cluster of lipid A corresponds to a region of the membrane with high positive curvature (∇^2^ *h* > 0) induced by the intrinsic curvature of lipid A, and clusters are separated by bands of high negative curvature (∇^2^ *h* < 0) which keep *h* small and minimize the pinning energy associated with separation of the membrane from the cell wall.

We can quantitatively account for lipid-cluster size as the optimal compromise between short-range attraction and long-range elastic repulsion. First, ignoring the elastic energy, the free energy gained by adding a like lipid to an existing cluster of *N* lipids tends toward a constant (linearly related to the perimeter of single lipid) times ɛ. In this case, for *ɛ* > *ɛ*_crit_, the free-energy minimum occurs at the maximal cluster size. The elastic potential, *V*(*r*), is shown in Figure 2H, along with a Gaussian fit with amplitude *α* = 0.28 *k_B_T* and width *σ* = 5.6 nm. These parameters are determined by the stiffness *κ* and pinning *λ,* with 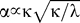 and 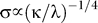 . Except at very short distances, the Gaussian is an extremely good fit (R^2^ > 0.99). The transition from a segregated phase consisting of one large cluster to the mixed phase of many small clusters occurs as a sharp transition with respect to the intrinsic curvature*γ,* and the preferred cluster size is then small with respect to the width of the elastic potential. We calculate the elastic free energy per lipid, due to both curvature and pinning, by integrating the elastic potential over a circular lipid cluster. The resulting repulsive elastic energy scales with the square of the lipid intrinsic curvature, ∼*γ* ^2^, and competes with the short-range attraction, ∼*ɛ* (see Materials and Methods). The minimum total free energy per lipid occurs at an intermediate cluster size. In Figure 2I, we plot the predicted and average cluster sizes for the sets of parameters in 2A–2F. The agreement indicates that neither the finite simulation cell nor cluster–cluster interactions significantly affect the optimal cluster size. Since our choice of parameters lies within the range of existing estimates for eukaryotic lipids [22,23], it is reasonable to expect the existence of lipid clusters on the order of 100–1,000 lipids in bacterial cell membranes.

### Lipid Clusters Stably Localize to the Cell Poles

**Figure 3:**
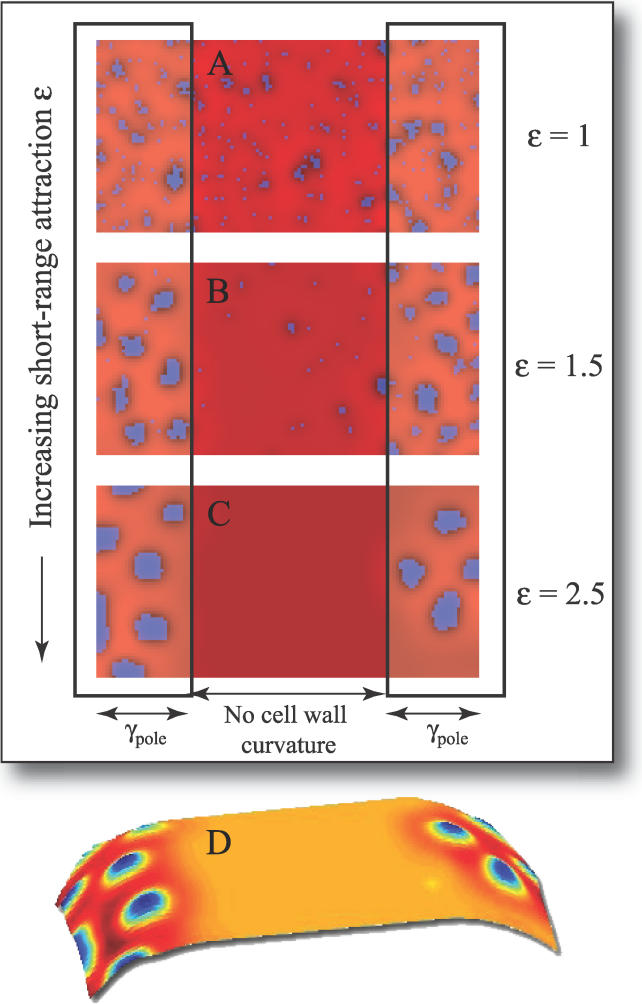
Polar Localization of Lipid Clusters Elastic energy parameters are fixed (*κ* = 25 *k_B_T, λ* = 0.25 *k_B_T*/nm^4^, *γ* = 0.4 nm^−1^), with varying short-range attraction (A) *ɛ* = 1 *k_B_T,* (B) *ɛ* = 1.5 *k_B_T,* and (C) *ɛ* = 2.5 *k_B_T*. The rectangles on the left and right represent the cell poles and have slightly enhanced cell-wall curvature, *γ*_pole_ = 0.04 nm^−1^. The three-dimensional figure in (D) represents the lipid position and the membrane height in (C) mapped onto the surface of a capped cylinder. The color scheme and fraction of lipid A (*ϕ* = 0.075) are the same as in Figure 2.

We next ask whether lipid clusters can spontaneously localize to bacterial cell poles. In Figure 3, we show the results of simulations in 60 × 120 cells (periodic in the vertical direction to represent a cylinder) in which the leftmost and rightmost quarters of the cell wall (the “poles”) have an underlying curvature of *γ*_pole_ = 0.04 nm^−1^ relative to the central cylindrical region. Using the same elastic parameters *κ* and *λ* and lipid parameters *γ* = 0.4 nm^−1^ and *ɛ* = 2.5 *k_B_T* as in Figure 2B, we show in Figure 3C that the difference in cell-wall curvature is sufficient to localize all of the clusters of lipid A to the “poles” of the cell. Importantly, this localization is critically dependent on cluster size. With these parameters, the polar localization energy of a single lipid with area *A*_lipid_ = l nm^2^ is only Δ*E*_pole_ = *κγγ*_pole_ *A*_lipid_ = 0.4 *k_B_T*. Using the polarly localized lipid configuration in Figure 3C as the initial lipid distribution, we reduce *ɛ* to 1.5 *k_B_T* in Figure 3B, and observe that, as lipid clusters shrink, the smaller clusters fail to localize to the cell poles. In Figure 3A, with *ɛ* further reduced to 1 *k_B_T*, lipids no longer cluster and are spread homogeneously throughout the membrane. Figure 3D is a visualization of the membrane height profile in Figure 3C mapped onto a section of cylinder with hemispherical endcaps.

Although in our simulations the cell-wall curvature *γ*_pole_ is only one-tenth the intrinsic curvature *γ* of lipids of type A, the polar localization of lipid clusters in Figure 3C can be easily explained within our energetic model. For a homogeneous cell wall with no underlying curvature, the predicted minimum in free energy per lipid occurs at a cluster size of 36 lipids with a total free energy 8 *k_B_T* higher than at the cell pole, where *γ*_pole_ = 0.04 nm^−1^, implying a thermal probability of 99.9% to find the cluster at the pole. (In fact, polar localization is even stronger if one takes into account the fact that the optimal cluster size grows to 47 lipids at the pole.) The energy change from moving a cluster of high-intrinsic-curvature lipids of type A from the lateral cylindrical region to one pole is −*N*Δ*E*_pole_, which scales linearly with the number of lipids, *N*, in the cluster. Our essential conclusion is that although the energy preference of an isolated lipid for the poles is only a fraction of the thermal energy *k_B_T, the cumulative energy gain due to reduced curvature mismatch for a lipid cluster is sufficient to result in stable polar localization*.

### Lipid-Cluster Sizes Insensitive to Membrane Composition

**Figure 4:**
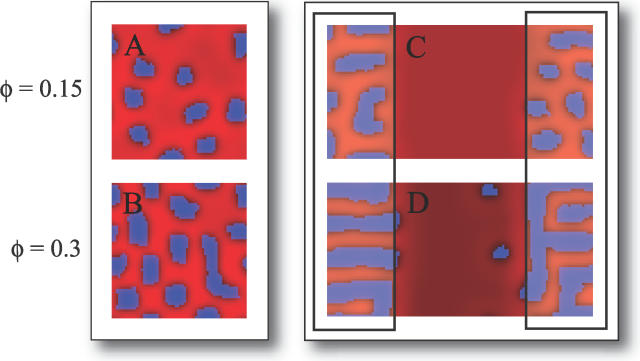
Effect of Membrane Composition on Cluster Formation (A,C) The percentage of lipid A is *ϕ* = 0.15; the clusters remain approximately the same size as in Figure 2B, with the same lipid and elastic parameters. (C) The rectangles at the left and right represent the poles of the cell with slightly higher curvature *γ*_pole_ = 0.04 nm^−1^, as in Figure 3. (B,D) Identical to (A) and (C), respectively, except that *ϕ* = 0.3.

Next, we consider the effects of varying the fraction *ϕ* of lipid A. For a cell of homogeneous curvature, we expect to find only small changes in cluster size as a function of *ϕ*, related to the weak interactions between lipid clusters. In Figure 4A, as *ϕ* is doubled from 0.075 to 0.15, the average cluster size in a 60 × 60 cell changes only slightly from 54 to 60. However, as the lipid A fraction *ϕ* is increased further to 0.3, other patterns start to appear. In Figure 4B, the lipids A begin to form long chains or stripes that are typical of cluster-forming systems at large filling fraction [24]. Similar effects are apparent in simulation cells with polar regions of higher curvature, as shown in Figure 4C and 4D.

### Lipid Clusters Stably Localize to Regions of Low Osmotic-Pressure Differential

**Figure 5:**
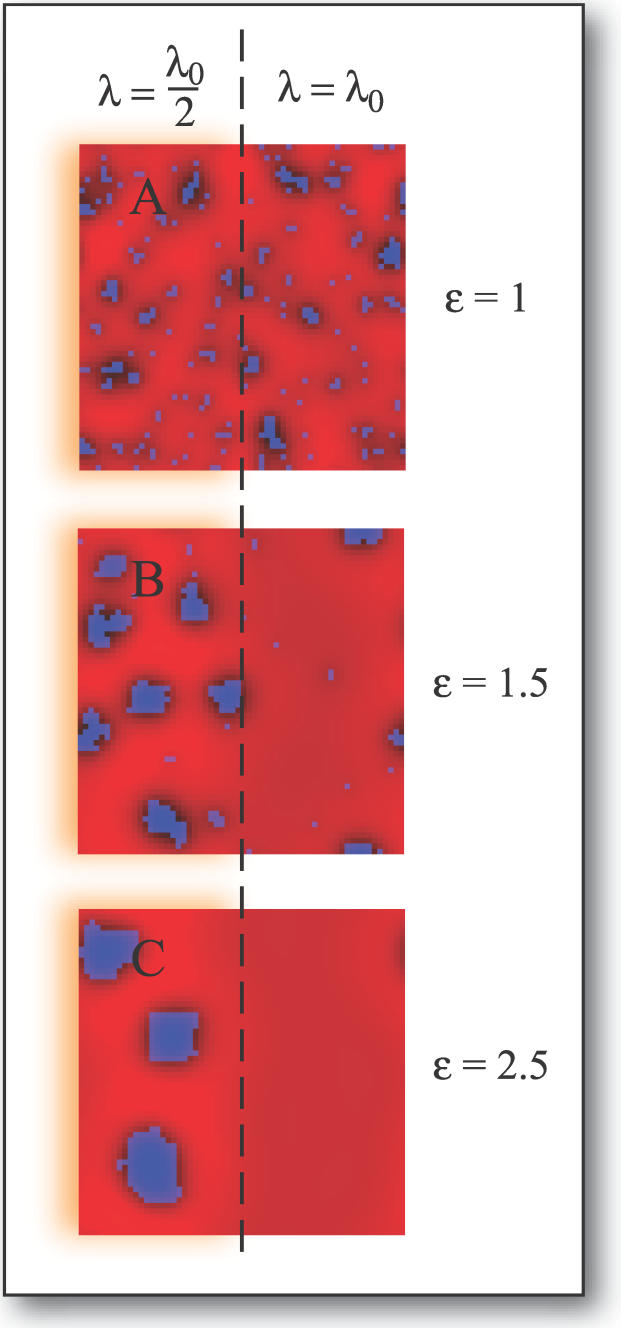
Localization of Lipid Clusters by Heterogeneous Membrane Pinning Membrane with uniform cell-wall curvature, fixed stiffness modulus *κ* = 25*k_B_T* and lipid A intrinsic curvature *γ* = 0.4 nm^−1^, and varying short-range attraction (A) *ɛ* = 1 *k_B_T,* (B) *ɛ* = 1.5 *k_B_T,* and (C) *ɛ* = 2.5 *k_B_T*. The pinning modulus of the left half of the membrane is lower by a factor of two (*λ* = *λ*_0_/2 = 0.125 *k_B_T*/nm^4^) relative to the right half of the cell (*λ* = *λ*_0_). The color scheme and fraction of lipid A (*ϕ* = 0.075) are the same as in Figure 2.

The strength of the membrane pinning modulus *λ* depends on the balance between the osmotic-pressure differential across the membrane and the inward force exerted by the cell wall. To mimic a heterogeneity in osmotic-pressure differential (e.g., for cells with a septal membrane), we vary the pinning modulus *λ* experienced by the membrane. In Figure 5, we show the results of simulations in 60 × 60 periodic cells in which the pinning modulus of the left half of the cell (*λ* = *λ*_0_/2 = 0.125 *k_B_T*/nm^4^) is lower by a factor of two relative to the right half of the cell (*λ* = *λ*_0_ = 0.25 *k_B_T*/nm^4^). Using the same stiffness modulus *κ* = 25 *k_B_T* and lipid parameters *γ* = 0.4 nm^−1^ and *ɛ* = 2.5 *k_B_T* as in Figure 2B, we show in Figure 5C that the difference in pinning modulus is sufficient to localize clusters of lipid A to the region mimicking low osmotic-pressure differential. As with localization due to variation in cell-wall curvature, this localization is critically dependent on cluster size. Using the configuration in Figure 5C as the initial lipid distribution, we reduced *ɛ* to 1.5 *k_B_T* in Figure 5B, and then to 1 *k_B_T* in Figure 5A, and observed progressive loss of localization as clusters shrink.

The localization of lipid clusters in Figure 5C to regions of low pinning modulus *λ* can be easily explained within our energetic model. Even a modest decrease in the pinning modulus increases the range and decreases the amplitude of the repulsive potential. For example, the factor of two decrease in *λ* in Figure 5 leads to an increase in the optimal cluster size from 36 to 42 and a decrease in free energy per lipid of 0.28 *k_B_T*. Even at the smaller cluster size, the total cluster energy is lowered by over 10 *k_B_T* by the decrease in the pinning modulus. Therefore, spatial variations in the strength of pinning by the cell wall can serve as a strong localization mechanism. If present, pinning heterogeneity can dominate over curvature, allowing lipid localization in either leaflet of the membrane.

## Discussion

**Figure 6:**
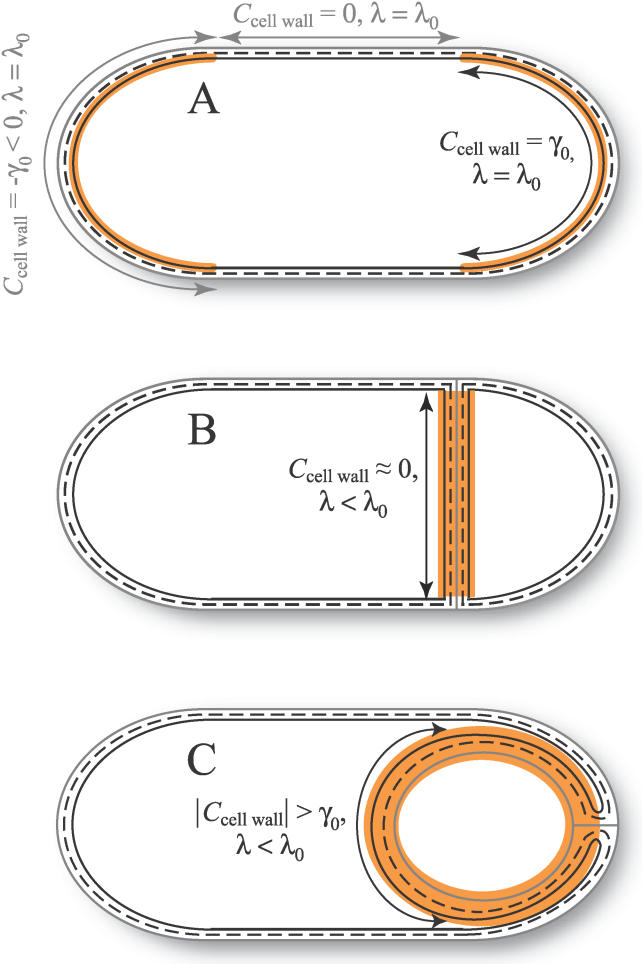
Competition between Curvature and Osmotic Localization of Lipid Clusters during Sporulation and Cell Division (A) During exponential growth, high-intrinsic-curvature lipid clusters localize to the poles of the inner leaflet (solid black curve), driven by differences in membrane curvature. All curvatures are shown relative to curvature in the cylindrical region of the cell. The cell-wall curvature experienced by the outer leaflet (solid gray curve) is opposite in sign to the intrinsic lipid curvature. Orange regions denote cardiolipin localization. (B) A lower osmotic-pressure differential across the septal/forespore-engulfing membrane corresponds to a reduced value of the pinning modulus *λ* and induces relocalization of the lipid clusters to the septal membrane (see Figure 5). (C) As the spore is engulfed, the clusters can migrate along the continuous leaflet consisting of the inner leaflet of the mother cell and the outer leaflet of the forespore-engulfing membrane to localize around the spore due to low osmotic-pressure differential.

Though anchors are a common source of subcellular protein segregation, ultimately these anchors themselves must be localized by some symmetry-breaking process. Therefore, it may be fruitful to consider possible mechanisms of spontaneous self-organization within cells. Here, we have demonstrated that the bacterial cell wall constrains the plasma membrane to produce finite-sized clusters of high-curvature lipids. These clusters are large enough to spontaneously and stably localize to the cell poles, and present in large enough numbers to achieve roughly uniform and equal coverage of both poles. Importantly, these results explain the recent observations of cardiolipin localization to both the poles and division site of rod-shaped bacterial cells, as assayed by NAO fluorescence (see Figure 6A).

Interestingly, cardiolipin localization to the forespore membrane during sporulation in B. subtilis is accompanied by an apparently complete loss of polar localization [9], as shown schematically in Figure 6. Though it is unlikely that the curvature of the forespore membrane is significantly higher than that of the poles, there should be little or no osmotic-pressure differential across the forespore membrane. As shown in Figure 5C, a modest decrease in osmotic-pressure differential that reduces the membrane-pinning modulus is enough to localize all of the cardiolipin to the region of membrane with low pressure differential (see Figure 6B). This effect can dominate over differences in membrane curvature. Therefore, our model naturally explains the relocalization of cardiolipin from the poles of the mother cell to the low-pressure forespore membrane observed experimentally [9] in B. subtilis cells early in sporulation. During engulfment of the forespore, the inner leaflet of the mother cell and outer leaflet of the engulfing membrane form a continuous monolayer. This allows the relocalization of cardiolipin from the pole of the mother cell first to the forespore septal membrane and then to the forespore-engulfing membrane, without requiring flipases to transfer cardiolipin between membrane leaflets (see Figure 6C).

The lipid-cluster sizes considered in this report are certainly below the resolution limit of conventional light microscopy, although they are similar to some estimates of lipid-raft dimensions [25], and recent developments in structural illumination [26] might allow for the observation of clusters with a radius as small as 50 nm. In addition, we note that we have chosen relatively large values for the intrinsic curvature of lipid A to result in computationally manageable cluster sizes; the predicted cluster size for *γ* = 0.04 nm^−1^ is ∼600. Regardless, our model for polar localization has important consequences.

First, we predict that cardiolipin clusters in rod-shaped cells will localize to the regions of highest curvature, namely the cell poles. As a corollary, we predict that spherical bacteria will not exhibit significant cardiolipin localization. Our model also explains the experimental observation that cardiolipin localizes to the division site of E. coli and B. subtilis [7–9], a region of low osmotic-pressure differential once the septum begins to close. Similarly, our model explains the localization of cardiolipin to the forespore membrane, a region of low osmotic-pressure differential, and predicts that changes in cardiolipin localization as osmotic-pressure differential is varied can be used experimentally to probe the strength of cell-wall pinning.

Second, as clusters partition binomially to the poles of rod-shaped cells, their mean separation is governed by a weak repulsive interaction between nearby clusters that, on average, produces a semiordered lattice of clusters. To distinguish our model of finite-sized cluster formation from large-scale domain formation driven by complete phase segregation, our model predicts that staining specific to at least one of the low-intrinsic-curvature phospholipids should reveal significant levels of fluorescence at the poles (overlaying the cardiolipin signal) and in minicells, arising from the interstitial regions between cardiolipin clusters in the inner leaflet and throughout the outer leaflet. Indeed, recent experiments using the phosphatidylethanolamine-specific cyclic peptide probe Ro09–0198 have demonstrated that significant levels of phosphatidylethanolamine are present along with cardiolipin at the poles of B. subtilis and E. coli cells [27].

Third, we expect that with in vivo control of the cardiolipin production through induction of the cardiolipin synthase, the NAO fluorescence should be uniform across the poles, on average, even with lower than wild-type levels of cardiolipin. The number of clusters in the lateral cylindrical region should remain low until cardiolipin levels are high enough that the poles become densely packed with clusters. Similar to Figure 4, subsequent additions of cardiolipin should produce clusters that distribute uniformly throughout the cylindrical region. This global increase in cardiolipin concentrations everywhere between the poles distinguishes our model from a scenario in which lipid localization is a direct consequence of the polar localization of cardiolipin synthase. We note in addition that the rapid diffusion of lipids in the membrane would likely eliminate any nonuniformities in concentration due solely to synthase localization.

Our model of cell-wall–mediated cluster formation is a robust mechanism for localizing and partitioning high-intrinsic-curvature lipids in equal amounts to both poles, and suggests that, similar to lipid rafts, the polar localization of cardiolipin may have important biological consequences for polar targeting of proteins. Although curvature cannot mediate stable polar localization of *individual* proteins for the same reason of length-scale mismatch given above for lipids, our model also suggests that some proteins, such as the chemotaxis receptors [28], may be localized to the poles via the aggregate curvature of large clusters.

## Materials and Methods

We represent the membrane as a two-dimensional square lattice fully occupied by lipids of two types, A and B, where lipid A is taken to have the higher intrinsic curvature. The intrinsic curvature of the lipids in a given spatial pattern determines the curvature function *C*(**r**). *C*(**r**) is measured relative to the cell-wall curvature, which may itself vary spatially (e.g., at the poles versus the lateral cylindrical region):


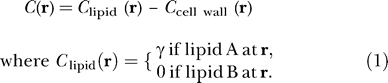
*C*(**r**) = *C*_lipid_ (**r**) – *C*_cell wall_ (**r**)


The height of the membrane *h*(**r**) is measured as the normal distance from the “unperturbed” position of the membrane, assumed to parallel the rigid cell wall, and *h*(**r**) may be positive or negative. The total energy of the membrane is a sum of two contributions: (i) the elastic energy

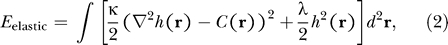
whose first term, proportional to the stiffness *κ,* penalizes mismatch in curvature between the membrane and the preferred intrinsic curvature of the lipids, and whose second term, proportional to the pinning modulus *λ,* penalizes deformations of the membrane away from the cell wall; and (ii) a short-range attractive interaction between like lipids

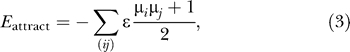
where the sum is over all pairs of nearest neighbors *i, j,* and *μ_i_* = 1 for lipids of type A and −1 for type B. We measure *κ* and *ɛ* in units of the thermal energy *k_B_T,* and *λ* in units of *k_B_T*/nm^4^. Although the surface area of a single lipid can vary due to tension and membrane bending, we assume for simplicity that all lipids occupy a 1 nm^2^ square, with the membrane height *h*(**r**) measured in nanometers.

We use a Metropolis Monte Carlo algorithm to minimize the energy of the membrane. Randomly selected pairs of lipids are exchanged with probability min 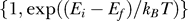 , where *E_i_* and *E_f_* are the total energy before and after the move, respectively. We use simulated annealing to escape local minima and guarantee a broad exploration of the energy landscape. All cluster sizes were computed as an average over ten Monte Carlo simulations with different random initial lipid configurations.

We note that with a stiffness modulus of *κ* = 25 *k_B_T,* the energy difference between polar and nonpolar localization for a lipid with intrinsic curvature *γ*_lipid_ = 0.1 nm^−1^ and cross-sectional area *A*_lipid_ = 1 nm^2^ in a rod-shaped cell with polar hemisphere diameter 1 *μ*m (corresponding to polar curvature *γ*_pole_ = 0.004 nm^−1^) is approximately *κγ*_pole_ *γ*_lipid_ *A*_lipid_ = 0.01 *k_B_T*.

## Supporting Information

### Accession Numbers

The primary protein accession numbers (in parentheses) from the Swiss-Prot databank (http://www.ebi.ac.uk/swissprot) for the proteins mentioned in the text are: Div J *C. Crescentus* DIVJ_CAUCR (Q03228), DivK *C. Crescentus* Q454976_CAUCR (Q45976), MinC *E. coli O157* MINC_ECO57 (Q8XDN0), MinD *E. coli O157* MIND_ECO57 (P0AEZ5), MinE *E. coli O157* MINE ECO57 (P0A736), MreB *C. Crescentus* Q9A821_CAUCR (P37894), and PleC *C. Crescentus* PLEC_CAUCR (P37894).
